# ‘Beyond GDP’ Requires Replacement, Not Just Additional Metrics

**DOI:** 10.1007/s11205-026-03837-5

**Published:** 2026-03-30

**Authors:** Jeroen van den Bergh

**Affiliations:** 1 Institute of Environmental Science and Technology, Universitat Autònoma de Barcelona, 08193 Bellaterra (Barcelona), Spain; 2 ICREA, Barcelona, Spain; 3 School of Business and Economics & Institute for Environmental Studies, Vrije Universiteit Amsterdam, Amsterdam, 08193 The Netherlands

**Keywords:** Growth, Equity, Environment, Sustainability, ISEW, GPI

## Abstract

Political decision-making remains dominated by GDP, often at the expense of social and environmental concerns. Indicator dashboards have so far had limited impact on core policy decisions in this regard. This article argues that only replacing GDP with a comprehensive aggregate metric can overcome its dominance. To illustrate which alternative is most likely to succeed, the concept of “perception distance” (or similarity) to GDP is introduced, formalized and compared across key options. Furthermore, it is argued that replacing GDP with a superior aggregate metric will enhance the effectiveness of the additive approach as the new headline metric reduces tension with disaggregate domain indicators for wellbeing, inequality and sustainability.

## Introduction

The continued focus on GDP in politics, media and society sits in the way of genuine progress and sustainability. Hence, we have embraced the idea of going beyond GDP. But it is little recognised that this involves a critical choice between two very different approaches: (1) an additive approach, or supplementing GDP with wellbeing and environmental indicators, giving rise to a dashboard; and (2) replacement of GDP, or substituting it as the central measure of progress by an alternative aggregate metric. This distinction is crucial. Unless it is clear whether ‘going beyond GDP’ means adding indicators or replacing GDP entirely, the phrase risks becoming a slogan that can justify different agendas.

An avalanche of recent publications promotes the dashboard approach, championed not only by institutions such as the UN and OECD, but also by initiatives like the Doughnut framework and a wide range of academic studies (Costanza et al., 2025). These tend to treat dashboards as a settled solution to the tension between economic growth and other societal goals. In this paper, I argue that this confidence is misplaced.

## Additive Approaches Have Not Overcome GDP Dominance

The additive approach is politically comfortable, which has contributed to it becoming the mainstream interpretation of “beyond GDP”. It allows policymakers to retain GDP while complementing it with additional indicators. The Stiglitz–Sen–Fitoussi Commission (Stiglitz et al., 2018), the OECD Wellbeing Dashboard (OECD, 2011), the Sustainable Development Goals, and various national wellbeing frameworks all take this route (Hoekstra, 2019).

Despite its popularity, there is no evidence that the additive approach has reduced GDP dominance. None of the existing dashboard initiatives has been able to challenge GDP’s dominant role in political discourse, media reporting and macroeconomic modelling. It is also unlikely that an additive strategy ever will achieve this: as long as GDP remains in place, ministers, journalists, and policymakers can too easily default to GDP growth as the headline signal. Adding indicators merely facilitates different actors to cherry-pick their preferred subset, in turn contributing to fragmentation – without weakening GDP’s gravitational pull. Many proposed additional indicators are important, such as life expectancy, education, crime, gender equality, housing, infrastructure, or air and water quality. But they have always coexisted with GDP, as have the traditional macroeconomic indicators of unemployment and inflation. At no point have they matched or threatened GDP’s primacy.

The additive approach is a dead end if the aim is to reduce GDP’s dominance. Dashboards inevitably invite subjectivity in political decision-making: selecting, prioritising, or weighting indicators often reflects personal or partisan preferences rather than societal needs. The set of 17 SDGs with 169 targets exemplifies this dynamic: national ministries, city departments, and public as well as private agencies highlight the SDGs that align with their scope or narrative while effectively assigning zero weight to the rest. For instance, ministries of economics tend to emphasise SDG 8, ministries of social affairs prioritise welfare-related SDGs (e.g., poverty, hunger, health or equality), and environmental ministries focus on sustainability-related SDGs (e.g., climate action, life below water or life on land). This illustrates that the SDGs tend to function as menus that foster compartmentalisation, whereas only an integrated approach could replace or compete with GDP.

Finally, by not replacing GDP, dashboards leave leeway for a green growth perspective. This is particularly evident in the SDGs as these retain economic growth as a core objective, notably through SDG 8, “Decent Work and Economic Growth”. This assumes that continued economic growth is compatible with other goals, including SDGs 12–15 that capture distinct dimensions of environmental sustainability.

## Advantages of the Replacement Approach

GDP’s power rests on its simplicity: a single, internationally comparable number that conveys economic progress at a glance. Dashboards and multi-indicator approaches cannot match this communicative force; they leave policymakers free to cherry-pick indicators while continuing to rely on GDP growth as the headline measure. By consolidating multiple dimensions of wellbeing, inequality, and environmental sustainability into a single metric, a replacement measure can retain the familiarity, comparability, and interpretability that make GDP politically and socially potent.

A replacement metric can thus serve as a clear signal of policy success in media and public debate. By keeping GDP’s communicative advantages while correcting its blind spots, a replacement metric can shift incentives, guide decisions toward sustainable prosperity, and ultimately dethrone GDP as the dominant measure of progress.

A further advantage is that a replacement metric automates socially desirable trade-offs by embedding societal costs and benefits within a single measure. Dashboards, by contrast, make trade-offs explicit and hence politically contentious. For example, the suggestion of sacrificing traditional GDP growth for improvements in health, safety, or climate stability will provoke political resistance, whereas in a corrected GDP this trade-off would be implicit. Overall, a *replacement* metric offers the best chance to move beyond the conventional GDP.

The UN High-level Expert Group on Beyond GDP is considering an adjusted GDP. Although its most recent report features the headline “An urgent need for a new compass”, it clarifies that “We are not recommending the replacement of GDP as a measure of economic activity. Our task is to complement and go beyond GDP.” (HLEG, 2025). In a related online lecture (HLEG, 2026), it was further suggested that an adjusted GDP would include corrections for wellbeing and equity, while sustainability would be separately measured – in effect resulting in a (small) dashboard. In practice, an adjusted GDP of this kind would still be likely to take precedence over sustainability indicators whenever trade-offs arise.

If the goal is truly to move beyond GDP, a more effective approach would be to construct a corrected GDP that integrates wellbeing, inequality, and core dimensions of environmental sustainability. This makes it much clearer when economic growth masks deeper problems. If conventional GDP or a welfare-adjusted GDP were to increase, but environmental unsustainability caused the fully adjusted metric to remain constant or decline, this would send a powerful signal that sustainability conditions and policies need to be strengthened.

## Monetary Aggregation Versus Subjective Weights

It is insufficiently recognised that monetary aggregation represents the most practical route for implementing a replacement metric. The principal alternative is the construction of a composite index. Yet many proponents of the latter overlook that foregoing monetary units does not eliminate valuation or value judgments (Costanza, 2024); it merely shifts the challenge to selecting and justifying weights for indicators with distinct units – an extremely difficult task.

A monetary approach has three main advantages. First, users of GDP are accustomed to monetary units, easing the transition away from it as the headline indicator. Second, monetary valuation removes the need for ad hoc and subjective (implicit or explicit) weights that characterise aggregation in composite indexes. Third, it can draw on a long history of well-established methods with numerous real-world applications, including to environmental, transport, health, energy, labour and urban issues (Hanley et al., 2002).

To illustrate why a non-monetary metric is unlikely to replace GDP, consider the Human Development Index. Despite its prominence in academic research and endorsement by the United Nations, it has never assumed the central role that GDP occupies. This is due to two fundamental limitations: unlike GDP, HDI is not a monetary measure; and it is explicitly bounded from above. By construction, HDI can thus not grow indefinitely but asymptotically approaches a maximum value. This ceiling follows from the fact that HDI is a dimensionless index defined on a scale from 0 to 1 – following from a rather arbitrary aggregation process – rather than an unbounded monetary measure. Because the index necessarily plateaus as countries develop, it conveys the politically inconvenient message that “progress” must eventually stop. For this reason, HDI has found little traction as a headline indicator in highly developed economies.

The strength of monetary valuation to construct better progress metrics is frequently underappreciated. Monetary valuation relies on elicitation mechanisms that leverages people’s familiarity with monetary trade-offs. These mechanisms include market prices, revealed and stated preferences (Champ et al., 2017), dose–response or production functions, anchoring happiness or subjective wellbeing to income (van Praag and Ferrer-i-Carbonell, 2004), and value transfer (Brouwer, 2000). Many of these methods further reflect respect for democratic decision-making by including the opinions of a representative sample of citizens using sophisticated elicitation mechanisms. A critique that inequality affects values can be mitigated by normalising values for income before calculating mean or median estimates (Gsottbauer et al., 2015). Finally, one can use multiple methods to triangulate complex valuation challenges (Whitehead et al., 2008).

By contrast, we lack comparable experience and empirical grounding for assigning weights to indicators expressed in different units. Weighting schemes behind composite indexes typically rely on ad hoc judgement, political bargaining, or just arbitrary assumptions – a problem that becomes more pronounced as the number of indicators increases. Whereas valuation methods typically get the order of magnitude right, as shown by comparisons between stated- and revealed-preference approaches, weights used in composite indices tend to be arbitrary and are likely to introduce substantial and larger biases.

## Two Types of Monetary Metrics

Monetary indicators fall into two main categories: flow measures like corrected GDP (Bleys & Whitby, 2015) and stock measures like genuine savings, total capital or comprehensive wealth (Pearce & Atkinson, 1993; Hamilton & Clemens, 1999; World Bank, 2024). Replacing GDP with an adjusted, corrected, “greened,” or sustainable GDP – which internalises environmental damage, resource depletion, informal economic activities (e.g., household labour) and inequality – is easier to understand and communicate than abstract wealth-based approaches. Moreover, by reducing the fixation on conventional GDP growth without becoming anti-growth, it would support a transition to an “agrowth” stance of indifference toward GDP growth (van den Bergh, 2017). Instead, stock measures would likely exist alongside GDP rather than replace it, as they differ fundamentally in nature and interpretation. In particular, total capital and comprehensive wealth tend to relate more directly to (un)sustainability – i.e. the (in)capacity to maintain wellbeing over time – than to current wellbeing itself.

Corrected GDP metrics translate different wellbeing components into a common monetary unit, thereby avoiding the need to impose subjective weighting schemes. The key point is not that money directly measures wellbeing, but that monetary valuation provides a familiar, empirically grounded language for aggregating diverse outcomes. One can even monetise inequality by using the Atkinson index, which compares actual mean income with the equally distributed equivalent income, showing how much social welfare expressed in income terms society effectively loses due to the unequal distribution (Atkinson, 1970).

## “Perception Distance” of Alternative Metrics from GDP

One way to assess the potential of alternative metrics to replace GDP is to measure their “perception distance” (or “cognitive-behavioural distance”) from GDP along three key dimensions: similarity of units (monetary vs. non-monetary), extent of aggregation (single vs. multiple indicators), and similarity of interpretation (income vs. other). These components capture how easily policymakers, media, and the public can adopt an alternative metric in place of GDP. Formally, the difficulty of replacement can be expressed as the sum of the distances along these three dimensions:

$$Perception\;distance\;to\;GDP\:=\:unit\;distance\:+\:aggregation\;distance\:+\:interpretation\;distance\\$$

Using a simple scoring scheme – assigning 0 to low distance and 1 to high distance^1^ – we can compare several prominent alternatives, including corrected GDP measures like the Index of Sustainable Economic Welfare (ISEW) and the Genuine Progress Indicator (GPI)^2^, another monetary approach such as Comprehensive Wealth, a composite metric like the Human Development Index (HDI), and dashboards with multiple indicators such as the Sustainable Development Goals (SDGs). The results, summarized in Table 1, illustrate that the dashboard approach exhibits the greatest overall distance from GDP, while corrected GDP measures display the smallest distance. In between are Comprehensive Wealth and HDI. The greater the overall distance, the more difficult it will arguably be for a beyond-GDP metric to serve as a functional replacement for GDP that can genuinely help to move beyond GDP dominance.

The table suggests that corrected GDP metrics, by remaining close to GDP in terms of units, aggregation, and interpretation, have the strongest potential to replace it as the primary headline indicator. In fact, a corrected GDP would yield a surrogate – a GDP 2.0 or GDP+ – that preserves many of GDP’s communicative strengths.

**Table 1:** Comparing “perception distance” to GDP for various beyond-GDP approaches

Beyond-GDP approach	Unit distance	Aggregation distance	Interpretation distance	Overall perception distance to GDP (sum of unit, aggregation & interpretation distances)
*Corrected GDP measures*	0	0	0	0
*Comprehensive wealth*	0	0	1	1
*Human Development Index*	1	0	1	2
*Sustainable Development Goals*	1	1	1	3

scores for “interpretation distance” are motivated by discussions in previous sections

These findings are complemented by other studies. A comparison of 65 beyond-GDP metrics shows that GPI is among the few approaches that capture wellbeing, equity and environmental sustainability (Jansen et al., 2024). Another study identifies the GPI as the most comprehensive aggregate alternative to GDP, directly linking to 14 of the 17 Sustainable Development Goals (Cook & Davíðsdóttir, 2021). Overall, there is much to say in favour of a beyond-GDP focus on a corrected GDP.

## A Sequential Strategy – Replacement Followed by Addition

We need broad consensus on a beyond-GDP approach. But consensus is only meaningful if the alternative can weaken GDP dominance. A proliferation of wellbeing indicators may enrich understanding but leaves the core narrative and political incentives to prioritise growth unchanged. As long as journalists and policymakers keep pointing to GDP as the definitive measure of success, supplementary indicators have little influence. Instead, a replacement metric allows one to say “There is a better measure – GDP 2.0; use that instead”.

A truly transformative beyond-GDP agenda requires taking the difficult but necessary step of replacing GDP with a superior headline measure. Doing so would realign political incentives, media reporting, and public perception, thereby steering policy decisions toward more inclusive and environmentally sustainable outcomes. Previously, I argued that the most effective approach would be to establish a dedicated panel under the auspices of the United Nations to carry out this task (van den Bergh, 2022). Such a panel should be mandated to assess suitable alternatives, test them with key stakeholders (citizens, journalists, politicians), and develop the corresponding accounting framework.

The current UN High-Level Expert Group on Beyond GDP represents an important starting point. It is to be hoped that it will take seriously the concerns raised here about what is required to genuinely dethrone GDP. UN involvement is essential, as replacing GDP must occur globally to address transboundary environmental challenges effectively. Only if all countries adopt a superior metric in place of GDP can the necessary policy changes gain broad support.

An easy way out is to resist this advice for replacement by emphasising that corrected GDP metrics are imperfect. But they would still be immensely better than the conventional GDP. Moreover, we already rely on imperfect alternative indicators, such as the HDI – which, in fact, has given rise to extended metrics correcting for inequality (IHDI) and planetary pressures (PHDI) (UNDP, 2025). Perhaps it is time for those who agree with this advice to join forces and, possibly in collaboration with the UN HLEG, develop a standardized fully-adjusted GDP approach, most likely in the spirit of ISEW/GPI.

It is important to note, finally, that replacing GDP does not mean abandoning the additive approach. On the contrary, once a better aggregate metric is in place, domain-specific indicators become far more effective because their tension with the headline measure is greatly reduced. The two approaches can then be combined in an integrated framework focused on improving the new aggregate metric over time while ensuring that dashboard indicators do not fall below agreed minimum thresholds. This in turn would allow societies to make clearer political choices on where to best position themselves on the broad spectrum from very weak to very strong sustainability (Fleurbaey & Blanchet, 2013). Such a configuration can guide societies most effectively toward sustainable prosperity.

## Data Availability

No data was used.

## Footnotes

1 Alternatively, one could frame the measure as a similarity rather than distance index. In that case, the scoring scale would be reversed, i.e. a small (large) distance would correspond to high (low) similarity. One could also add a fourth element reflecting similarity of components (e.g., if GDP or something similar like income or production is covered).

2 The Index of Sustainable Economic Welfare (ISEW) was developed by Daly and Cobb (1989) as an alternative to GDP, measuring welfare rather than total economic activity. It accounts for unpaid work, adjusts for income inequality, and deducts for defensive expenditures, environmental degradation, and depletion of natural capital. The ISEW metric extended the corrected GDP (“Measure of Economic Welfare”) of Nordhaus and Tobin (1972); during the 1990s the ISEW was further developed by incorporating additional adjustments for crime, unemployment and leisure time, and methodologically standardised, giving rise to the Genuine Progress Indicator (GPI). In essence, the GPI can be seen as a refined and more policy-oriented version of the ISEW. Details on their methods and applications are provided in Posner and Costanza (2011) and Malinauskaite et al. (2026).
